# Simultaneous real-time EEG-fMRI neurofeedback: A systematic review

**DOI:** 10.3389/fnhum.2023.1123014

**Published:** 2023-03-31

**Authors:** Giuseppina Ciccarelli, Giovanni Federico, Giulia Mele, Angelica Di Cecca, Miriana Migliaccio, Ciro Rosario Ilardi, Vincenzo Alfano, Marco Salvatore, Carlo Cavaliere

**Affiliations:** IRCCS SYNLAB SDN S.p.A., Naples, Italy

**Keywords:** EEG-fMRI, neurofeedback, simultaneous, biofeedback, clinical neuroscience

## Abstract

Neurofeedback (NF) is a biofeedback technique that teaches individuals self-control of brain functions by measuring brain activations and providing an online feedback signal to modify emotional, cognitive, and behavioral functions. NF approaches typically rely on a single modality, such as electroencephalography (EEG-NF) or a brain imaging technique, such as functional magnetic resonance imaging (fMRI-NF). The introduction of simultaneous EEG-fMRI tools has opened up the possibility of combining the high temporal resolution of EEG with the high spatial resolution of fMRI, thereby increasing the accuracy of NF. However, only a few studies have actively combined both techniques. In this study, we conducted a systematic review of EEG-fMRI-NF studies (N = 17) to identify the potential and effectiveness of this non-invasive treatment for neurological conditions. The systematic review revealed a lack of homogeneity among the studies, including sample sizes, acquisition methods in terms of simultaneity of the two procedures (unimodal EEG-NF and fMRI-NF), therapeutic targets field, and the number of sessions. Indeed, because most studies are based on a single session of NF, it is difficult to draw any conclusions regarding the therapeutic efficacy of NF. Therefore, further research is needed to fully understand non-clinical and clinical potential of EEG-fMRI-NF.

## 1. Introduction

In the late 1970s, Sterman showed evidence of an electroencephalogram (EEG) response to cold pressor stimulation, thus highlighting a feedback function in regulating electrical cerebral reactivity EEG rhythms (Sterman et al., 1966). Such an EEG response derives from the intrinsic electrical activity generated by the excitation and inhibition of pyramidal neurons (Lorente De Nó, 1947). Physiological processes, such as sleep state or progression to sleep, may produce changes in EEG frequency patterns, as well as motor and cognitive behaviors, leading, for example, to what is known as “alpha desynchronization” (Klimesch et al., 1998). Based on Pavlov's experiments, Sterman et al. (1966) used operant conditioning in opposition to classical conditioning (Staddon and Cerutti, 2003) to voluntarily modulate EEG activity in response to a sensory stimulus event or cognitive task. The resulting EEG's change in response to an external event, namely EEG feedback, could be an increase or a decrease in the activity, and it is respectively called “event-related desynchronization” or “event-related synchronization”. The first rhythmic EEG pattern identified was the sensorimotor rhythm (SMR), a particular brain rhythm in the range of 12–15 Hz.

Initially, EEG-biofeedback studies were done on experimental animals, then, this research was translated to humans, aiming at regulating SMR in uncontrolled epilepsy to reduce motor seizure rates (Sterman, 2000). In Attention Deficit Disorder and Hyperactivity (ADHD), EEG-biofeedback may improve the clinical pattern, reducing hyperactivity and enhancing attention (Lubar and Shouse, 1976). In anxiety, protocols involving the upregulation of the theta/alpha ratio were used to enhance the state of relaxation (Budzynski, 1999). Throughout the years, EEG-biofeedback was branded as EEG-neurofeedback (EEG-NF), and different protocols and approaches have been validated, such as SMR and SMR ratio, alpha/theta ratio, frontal alpha training, etc. (Gruzelier, 2014). These pioneering studies highlight the opportunity to volitionally control human electrocortical activity, modifying cognitive and motor functions in health and disease. Groppe et al. described the associations between the typical EEG rhythms and their functions, i.e., alpha rhythm is mainly related to attention and cognitive functions at all ages, theta rhythm to working memory load, and low beta to inhibition of sensorimotor cortex (Groppe et al., 2013).

NF has emerged as an alternative non-pharmacological treatment for several neurological and behavioral disorders. With the EEG digitalization in the 1990s and the advent of brain-computer interface (BCI), easier access to electrophysiological information was enabled by decoding brain activation patterns generated by NF tasks, thus increasing the use of the EEG-NF in neurology and psychiatry. The EEG is non-invasive, inexpensive, portable, and benefits from a high temporal resolution. However, EEG is very sensitive to noises, such as environmental components, or movements. Also, it lacks specificity because of its low spatial resolution (order of the centimeters) (Iannaccone et al., 2015). The introduction of BCI was fostered by its application to functional magnetic resonance imaging (fMRI): the fMRI-NF was introduced for the first time by Weiskopf et al. (2004).

In fMRI-NF, blood oxygenation level-dependent activity (BOLD), which is related to the vascular response to neural activity, is directly fed back to participants in the magnetic scanner. Participants are trained to voluntarily modulate this activity, increasing or decreasing it. Such training targets specific regions of interest (ROI) and modulates motor behavior, attention, working memory, and emotional and cognitive processes. For instance, the upregulation of BOLD activity of the primary motor region and amygdala is associated with improved motor performance and emotional functions (anxiety, stress, depression, etc.), respectively (Zotev et al., 2011).

Albeit fMRI brings significant added value to NF protocols, along with BCIs, many researchers still debate the real usefulness of fMRI-NF in clinical contexts. A concern is the amount of physiological noise and artifacts in the magnetic scanner, whilst a key advantage is that fMRI-NF provides feedback about the whole brain's ongoing neural activation, allowing for a spatial resolution in the range of millimeters. The BOLD correlation between two or more neural networks is called “functional connectivity”, NF can regulate the connectivity, and it may lead to long-term effects. The neural mechanisms engaged by neurofeedback and how they affect learning still need elucidation (Sulzer et al., 2013). These mechanisms involve wide and complex brain networks, including basal ganglia, temporoparietal areas, anterior cingulate cortex (ACC), and the anterior insula. These brain regions appear to be activated during the NF training regardless of the ROI targeted, hence questioning if their activation reflects the actual learning processes (Emmert et al., 2016).

Recently associations between electrophysiological and hemodynamic signatures of fMRI demonstrated their usefulness for diagnostic purposes (Marchitelli et al., 2016; Mele et al., 2019), overcoming the lack of specificity of the EEG, hence opening new insights in the field of neuromodulation. This correlation between the EEG and BOLD activity encouraged simultaneous EEG-fMRI-NF, providing a novel approach in neuroscience. Studying brain plasticity and reorganization through the continuous self-regulation training of specific brain areas allows for restoring lost neurological functions (Dewiputri and Auer, 2013). The self-regulation training process during EEG-fMRI-NF requires participants to modulate two different types of signals, driving back two feedbacks. How the feedback is represented may vary, as a function of stimulation type (e.g., verbal, visual, auditory, olfactory, or a combination of these, for instance, a size/height of a bar or changes in tones). In the literature, the application of either EEG-NF or fMRI-NF as a therapeutic tool in clinical populations suffering from neurological and psychiatric disorders is documented (Lofthouse et al., 2012; Arns et al., 2017).

EEG-fMRI-NF has also been applied to healthy subjects to boost and improve their cognitive and behavioral functioning (Gruzelier, 2014). However, the assessment of such a novel and promising procedure remains quite unexplored in the literature (but see Zotev et al., 2014), and to the best of our knowledge, there is no systematic review assessing the use of simultaneous EEG-fMRI-NF. Therefore, this study aims to summarize scientific evidence about the simultaneous EEG-fMRI-NF, highlighting experimental protocols, fields of application, and clinical effectiveness.

## 2. Material and methods

### 2.1. Search strategy and study selection

**Figure 1:**
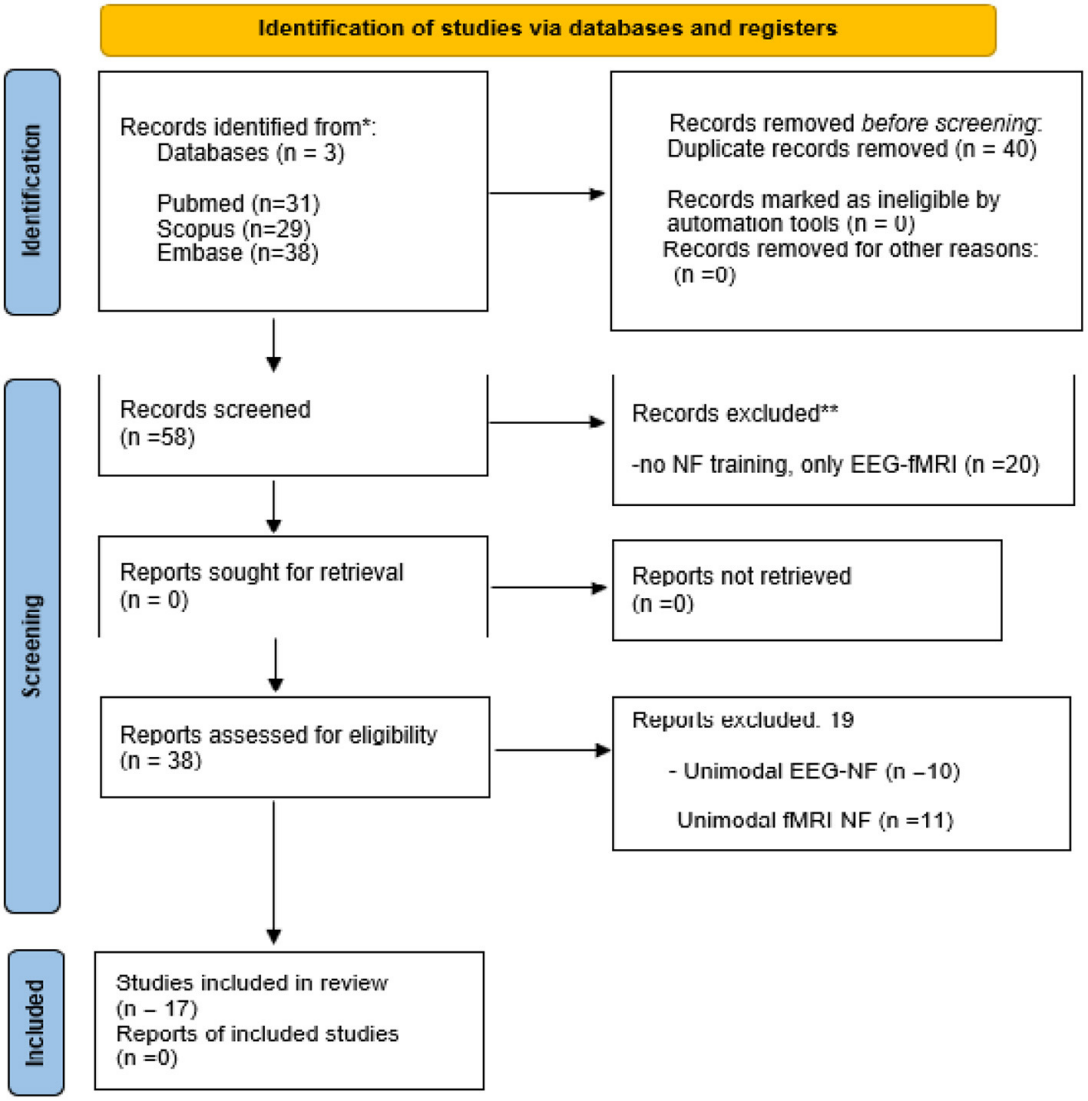
PRISMA study selection diagram. The diagram moves through the four stages of PRISMA study selection: identification screening, screening, eligibility and included. From an original count of 98 studies, 17 were finally selected for this review. NF, neurofeedback; EEG, electroencephalograph; fMRI, functional magnetic resonance imaging.

We searched PubMed, Scopus, and Embase according to the recommendation of the preferred reporting items for systematic reviews and meta-analysis (PRISMA) guidelines. We used the following keywords combined with the “AND” logic operator: EEG, fMRI and neurofeedback. Only English-language articles were included in the search. A total of 98 articles were found until May 2022 (first record: 2014). No time-frame window was set due to the shortfall of published research. When selecting literature, inclusion criteria were the simultaneity of the EEG and fMRI and the presence of NF training. The exclusion criteria were using one single modality with NF training (only EEG-NF or fMRI-NF), duplicate studies, the absence of NF training, and the use of NF without real-time EEG or fMRI, pre-prints, symposium presentations and book issues. The resulting articles were selected or rejected based on the criteria described in Figure 1.

### 2.2. Data collection process and extraction

Papers' titles were evaluated by two-independent operators to assess the eligibility of the selected papers; a third evaluator was included in cases of discordant assessment. When the inclusion or exclusion criteria were not met, also the abstract was included in the assessment. Finally, full-text screening was performed on the remaining papers to identify those that were not matched the eligibility requirements. In the tables, the studies are listed in chronological order. Table 1 includes studies that use bimodal EEG-fMRI-NF; Table 2 includes studies with unimodal fMRI or unimodal EEG NF with respectively passive simultaneous EEG and fMRI. The characteristics of the included studies were extracted according to the first author, aim of the study, sample, methods, protocol, results, and conclusion.

**Table 1:** Ten EEG-fMRI-NF studies included in the review, organized by: authors, aim of the study, sample, methods, NF protocol, neurophysiological results and behavioral results.

**References**	**Aim**	**Sample**	**Methods**	**NF protocol**	**Neurophysiological results**	**Behavioral results**
Zotev et al. (2014)	Modulating emotional self-regulation	6 HC	32ch BrainAmp MR EEG	NF conditions: induce happy memories, count backwards subtracting a given integer, rest and relax	Participants simultaneously regulated their BOLD fMRI activation in the left amygdala and frontal EEG power asymmetry in the high-beta band	Simultaneous EEG-fMRI-NF training of amygdala is accompanied by decreases in state measure of depression
		No sham control	General Electric MR750 3T	1 session—~1 h		
				7 runs–7 min 40 s		
				Happy emotion induction task		
				Visual feedback: target bars		
Perronnet et al. (2017)	EEG-fMRI-NF where participants perform a motor-imagery task in unimodal and bimodal NF conditions	10 HC	64ch BrainAmp MR EEG	NF conditions:	Participants simultaneously regulated activity in the motor regions feedback from frontal EEG asymmetry in the beta band and from left amygdala BOLD	These results did not allow to get any preliminary insight into direct NF clinical effects due to small sample and only 1 single session of training
		No sham control	Siemens MRI 3T	A, unimodal EEG-NF B, unimodal fMRI-NF C, bimodal EEG-fMRI NF		
				1 session—~3 h		
				6 runs: Localizer (5.20 min), MI without NF (3.20 min), 3 NF runs with different NF conditions (6.40 min), post MI run without NF (3.20 min)—~30 min each run		
				Motor imagery task		
				Visual feedback: a moving ball on the screen		
Mano et al. (2017)	Describe how to set up a hybrid EEG-fMRI platform to conduct bimodal NF	n/a	n/a	n/a	The platform provides a pipeline of simultaneous acquisition integrating both modalities at all processing stages	This hybrid EEG and fMRI bimodal platform has been successfully used in two experimental trials with more than 100 NF sessions and more than 30 subjects
Savelov et al. (2019)	Bimodal NF training movement region (Brodmann area 4) in stroke patients for rehabilitation purposes	12 stroke patients	32ch BrainAmp MR EEG	NF conditions: work (clench or unclench wrist), rest	Participants modulated fMRI and EEG signals causing interesting, but not specified, cerebral dynamics of both methods	Clinical results were manifested by improvement of motor indices
		No sham Control	Ingenia Philips 3T	4 sessions—~1.20 h each		
				8 runs-−10 min each		
				Motor task		
				Visual feedback: circular metaphor		
Zotev et al. (2020)	Applying bimodal NF training in MDD to self-regulate emotion	24 MDD	32chBrainAmp MR EEG	NF conditions: induce happy memories, count backwords subtracting a given integer, rest and relax	Participants showed significant upregulation of the LA BOLD activity, FAA and FBA	Patients showed significant improvements: POMS, depression, confusion, total mood disturbance, STAI, state anxiety, VAS, happiness
		Unmedicated	General Electric MR750 3T	1 session—~50 min		
		Test 16 vs. Sham 8		6 runs-−8.40 min each		
		Single blind		Happy emotion induction task		
				Visual feedback: target bars		
Zotev et al. (2020)	Applying the exact low resolution brain electromagnetic tomography (eLORETA) to investigate EEG sources in the prefrontal regions and left amygdala using motor imagery	24 MDD	32chBrainAmp MR EEG	NF conditions: induce happy memories, count backwords subtracting a given integer, rest and relax	eLORETA shows positive upper alpha and high beta changes in the left prefrontal cortical regions, correlating with LA BOLD activations	Patients with higher anhedonia demonstrates larger reductions in UAD. Patients with higher anxiety shows reductions in HBD
		Unmedicated	General Electric MR750 3T	1 session—~50 min		
		Test 16 vs. Sham 8		6 runs-−8.40 min each		
		Single blind		Happy emotion induction task		
				Visual feedback: target bars		
Lioi et al. (2020a)	A pilot study in chronic stroke patients using multi-target motor imagery (MI)	4 stroke patients	64ch BrainAmp MR EEG	NF conditions: rest, work (MI), rest	Patients managed to upregulate the BOLD activity in the targeted motor areas	2 patients with intact CST improved their clinical FMA-UE score (upper limb motor function scale). Hence the success in upregulating target motor areas depends on severity of stroke damage
		No sham control	Siemens 3T	5 sessions (2 bimodal and 3 unimodal EEG-NF)	during NF training. The EEG activity was harder to modulate during bimodal sessions, but all patients successfully upregulated the activity recorded at motor electrodes during unimodal EEG-NF sessions	
				3 runs-−5.20 min each		
				Motor imagery task		
				Visual feedback: a ball		
Lioi et al. (2020b)	Describing a multimodal dataset of EEG-fMRI-NF acquired during a motor imagery task previously by Perronnet et al. (2017)	30 HC	64chBrainAmp MR EEG	NF conditions: rest, work (MI), rest	This dataset deepens the coupling model underlying the EEG and fMRI NF signal, advancing the methodologies for multimodal data integration and also EEG de-noising methods	This represents the first open access bi-modal NF dataset integrating EEG and fMRI
		No sham control	Siemens 3T	5 sessions—~30 min each		
				6 runs-−5.20 min each		
				Motor imagery task		
				Visual feedback: a ball		
Cury et al. (2020)	Machine learning: a model able to predict fMRI-NF scores learning from EEG-fMRI NF	17 HC	64ch BrainAmp MR EEG	NF conditions: rest, work (MI), rest	Different NF predictors were tested for each of the three learning sessions and for all subjects and the relation between EEG and fMRI changes over sessions	The Laplacian model appears to be the best solution. It could overcome the absence of fMRI-NF allowing the estimation of fMRI-NF scores when using only EEG to simplify its use in clinical settings
		No sham Control	Siemens 3T	3 sessions: 1 as learning and cross-validation step, 2 as testing step**—**5 min each		
		(Exiting dataset Perronnet et al., 2018)		Motor imagery task		
				Visual feedback: a ball		
Bezmaternykh et al. (2021)	EEG-fMRI NF and fMRI-NF training in Post-Stroke Motor Rehabilitation	Stroke patients	MR scanner Not specified	NF conditions: rest, work (MI), rest	Patient 1(fMRI-NF) increased the fMRI signal in target areas (premotor cortex and SMA). Patient 2 (fMRI-EEG) shows reduction in beta and mu EEG activity	Both patients showed clinical improvement in hand grip strength and speed of the movement
		No sham control	Brain Products	6 sessions—~20 min each-−8 runs (runs duration not specified)		
			MR EEG system	Motor imagery task		
				Visual feedback: type not specified		

HC, health control; EEG, electroencephalogram; fMRI, functional magnetic resonance imaging; NF, neurofeedback; BOLD, blood oxygenation level dependent; MR, magnetic resonance; I = motor imagery; MDD, major depression disease; EG, experimental group; LA, left amygdala; FAA, frontal alpha asymmetry; FBA, frontal beta asymmetry; POMS, profile of mood state; STAI, state-trait anxiety inventory; VAS, visual analog scales; eLORETA, exact low resolution brain electromagnetic tomography; UAD, upper alpha density; HBD, high beta density; CST, corticospinal tract; FMA-UE, Fugl-Meyer assessment of motor recovery; SMA, supplementary motor area.

**Table 2:** Seven unimodal studies (EEG-NF with simultaneous fMRI-NF or vice versa) included in the review organized by: authors, aim of the study, sample, methods, NF protocol, neurophysiological results, and behavioral results.

**References**	**Aim**	**Sample**	**Methods**	**NF protocol**	**Neurophysiological results**	**Behavioral results**
Zich et al. (2015)	Characterizing the relationship between motor imagery (MI) EEG-NF and concurrent fMRI activation in cortical sensorimotor areas	24 HC	EEG-NF with simultaneous fMRI	NF conditions:1 motor execution and three for MI	The contralateral MI related decrease in EEG sensorimotor rhythm amplitude correlated inversely with fMRI activation in the contralateral sensorimotor areas, whereas a lateralized fMRI pattern did not necessarily go along with a lateralized EEG pattern	MI-EEG signals and sensorimotor cortical activity are both similarly modulated by EEG-NF. This supports potential of MI EEG-NF for motor rehabilitation
		No sham Control	61ch BrainAmp MR EEG	2 sessions (6-week interval): 1 with simultaneous fMRI, 1 without fMRI—~44 min each		
			Siemens MRI 3T	4 runs-−11 min each		
				Motor task		
				Visual feedback: target bars		
Meir-Hasson et al. (2016)	Creating a common EEG model from one EEG electrode (cEFP model) to predict BOLD of deep brain region activity (amygdala) and integrating it with NF training	13 HC	EEG-NF with simultaneous fMRI	NF conditions: rest, modulate the sound volume	Amygdala BOLD activity was detected with cEFP. This was done by applying machine learning algorithms on EEG data acquired simultaneously with fMRI	The current framework demonstrated the potential of developing an EEG based model of localized BOLD activity to use in a range of clinical conditions such as PTSD
		Test 7 vs. 6 sham	30ch BrainAmp MR EEG	1 session—~35 min	EEG-EFP-NF modulated subjects' theta/alpha power ratio enhancing state of deep relaxation	
		Single blind	MRI scanner not specified	5 runs (first was baseline)-−7 min each		
				Relaxation task with eyes closure lowering the sound volume		
				Auditory feedback: sound		
Zotev et al. (2016)	Exploring correlations between fMRI-NF and simultaneous frontal EEG asymmetries (FEA)	24 MDD	fMRI-NF with simultaneous passive EEG	NF conditions: induce happy memories, count backwords subtracting a given integer, rest and relax	Individual amygdala's BOLD activity positively correlates with upper alpha EEG asymmetries (FEA) changes	Findings demonstrate positive correlation with the MDD patients' depression severity ratings and total mood disturbance hence being beneficial for MDD disease
		Test 13 vs. 11 sham	32ch BrainAmp MR EEG	2 sessions—~1 h		
		Single blind	General Electric Discovery	7 runs-−8.46 min each		
				Visual feedback: target bars		
Zotev et al. (2018a)	Regulating BOLD activity of mediodorsal (MD) and anterior thalamic nuclei (AN) and correlation with simultaneous EEG alpha rhythm	29 HC	fMRI-NF with simultaneous passive EEG	NF conditions: induce happy memories, count backwords subtracting a given integer, rest and relax	Findings shows significant increase in the MD BOLD activity and positive correlation with posterior alpha EEG power. Hence EEG-NF modulating alpha would complement fMRI-NF targeting MD and AN	As the parieto-occipital sources of alpha rhythm have been implicated in cognitive and memory functions, fMRI-NF can be used in memory function training that can be relevant in neuropsychiatric disorders
		Test 15 vs. 14 sham	32ch BrainAmp MR EEG	1 session—~1 h		
		Single blind	General Electric Discovery	7 runs-−8.46 min each		
				Visual feedback: target bars		
Keynan et al. (2019)	Using the EEG model Amygdala-EFP-NF for stress resilience and amygdala-related emotion regulation processes	180 HC (combat soldiers)	EEG-EFP-NF, post-training fMRI	NF conditions: attend the virtual scenario, regulate the scenario, washout, tapping fingers	Amyg-EFP-NF and control-NF showed a similar pattern of increased Amyg-EFP downregulation until the third session. The specificity of Amyg-EFP-NF is evident in sessions 4–6, demonstrating the importance of repeated NF sessions to achieve specificity	These results demonstrate limbic specificity and efficacy of Amyg-EFP-NF during a stressful period as it led to reduced alexithymia and faster emotional Stroop indicating better stress coping
		NF n = 45	32ch BrainAmp MR EEG	6 sessions-−1 or 2 sessions per week—~1 h each		
		No NF n = 45	**S**iemens 3T	6 runs—around 10 min each		
		EFP-NF n = 90		Visual feedback: 3D virtual hospital waiting room		
		Double blind		Happy emotion induction task		
Zotev et al. (2018a)	Upregulating BOLD activity in the left amygdala-prefrontal interactions in patients with PTSD	23 PTSD	fMRI-NF with simultaneous passive EEG	NF conditions: induce happy memories, count backwords subtracting a given integer, rest and relax	Left prefrontal amygdala BOLD activity was upregulated (EG>CG)	80% of the EG participants demonstrated clinically meaningful reductions in Clinician-Administered PTSD Scale (CAPS) ratings, compared to 38% in the CG. They also exhibited a significant reduction in comorbid depression severity
		Test 15 vs. Sham 8	32ch BrainAmp MR EEG	3 sessions—~1 h each	EEG coherence analysis shows correlation between upper alpha band and PTSD severity	
		Single blind	General Electric Discovery	7 runs-−8.46 min each		
			MR750 3T	Happy emotion induction task		
				Visual feedback: target bars		
Simões et al. (2020)	Reconstructing the BOLD-fMRI signal measured at the facial expressions processing network (FEPN) from EEG-NF training	10 HC	fMRI-NF with simultaneous passive EEG	NF conditions: facial expression, neutral and motion	The FeatPoolICA source model is created extracting EEG features for approximating the BOLD signal from FEPN. It has been compared across folds and subjects and it shows an accuracy of 56% and an improvement of 36% compared to the EFP model	The proposed FeatPoolICA BOLD approximation model may positively impact the transfer of fMRI-based neurofeedback interventions to EEG setups
		No sham Control	64chBrainAmp MR EEG	1 session-−35.33 min		
			Siemens 3T	4 runs: localiser (5.33 min) + 3 NF runs (10 min each)		
				Happy emotion induction task		
				Visual and auditory feedback (Intensity level of the facial expression displayed by the virtual avatar and high and low pitch beep sounds)		

Three studies use unimodal EEG-NF with simultaneous passive fMRI; four studies use fMRI-NF with simultaneous passive EEG recording.

HC, health control; EEG, electroencephalogram; fMRI, functional magnetic resonance imaging; NF, neurofeedback; BOLD, blood oxygenation level dependent; MR, magnetic resonance; MI, motor imagery; EFP, EEG fingerprint; FEA, frontal EEG asymmetry; MDD, major depression disease; EG, experimental group; PTSD, post-traumatic stress disorder; CG, control group.

## 3. Results

### 3.1. Study selection

**Figure 2:**
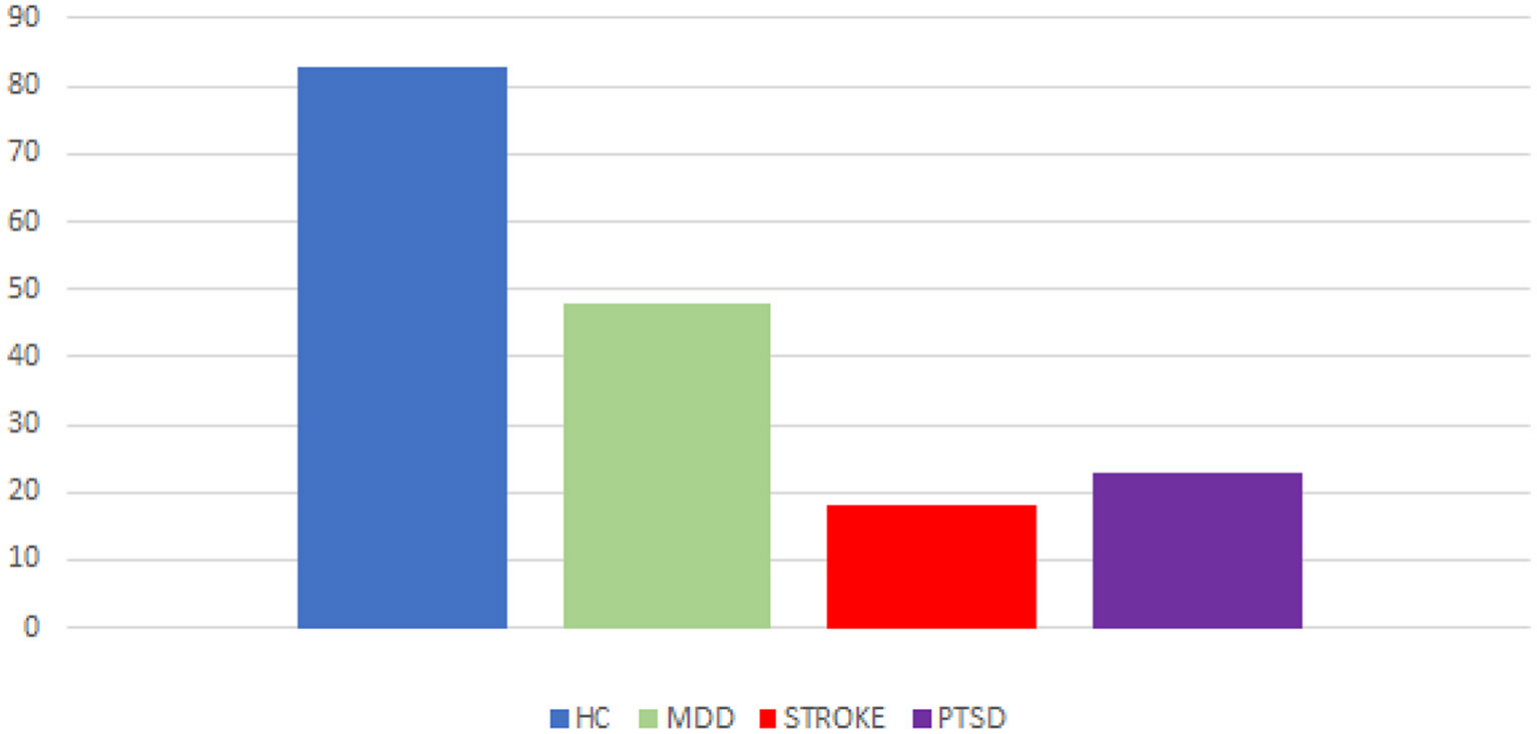
Proportion of EEG-fMRI-NF studies. Number of studies in relation to neuropsychological diseases (MDD, stroke, PTSD) and healthy control subjects.

A total of 98 articles were retrieved from the PubMed (N = 31), Scopus (N = 29), and Embase (N = 38) databases. After duplicate removal (N = 40), the remaining 58 papers were screened based on titles and abstracts. Of these 58 articles, 20 records were excluded because they did not involve neurofeedback training but only simultaneous EEG-fMRI technique. The remaining 38 articles were then read in full and assessed for eligibility, and 19 were then excluded: 10 because they focused on unimodal EEG-NF, 11 because they evaluated unimodal fMRI-NF. Ultimately, 18 records were included for qualitative analysis in this review. The PRISMA flow diagram describes the process of choice of the studies (Figure 1), and their characteristics are summarized in Tables 1, 2, for EEG-fMRI-NF studies with bimodal or unimodal feedback, respectively (Figure 2). Ten studies investigated the use of simultaneous acquired EEG and fMRI in a bimodal neurofeedback acquisition (Table 1). Simultaneous bimodal EEG-fMRI studies estimated the real-time NF from both signals. This means that EEG was recorded in the MRI scanner with an MR-compatible EEG cap in one or more sessions during fMRI acquisition. Participants were required to execute a task and modulate BOLD and EEG activities. Following a real-time quality pre-processing of BOLD and EEG, the NF was exported to a custom-made visualization software and shown to subjects using either a video or a display screen. Seven studies used the NF training only through one single modality (or fMRI-NF or EEG-NF), but both modality measures are simultaneously acquired for offline analysis (Table 2). Acquiring EEG synchronously with fMRI-NF allowed the detection of cerebral electrical activity features that are related to activations of BOLD activity. NF training may use EEG-correlates of fMRI responses instead of the expensive and complex MRI techniques, thus enabling the NF application in any clinical setting using the more affordable EEG (Formaggio et al., 2010).

### 3.2. Multimodal EEG-fMRI-NF on healthy participants

**Figure 3:**
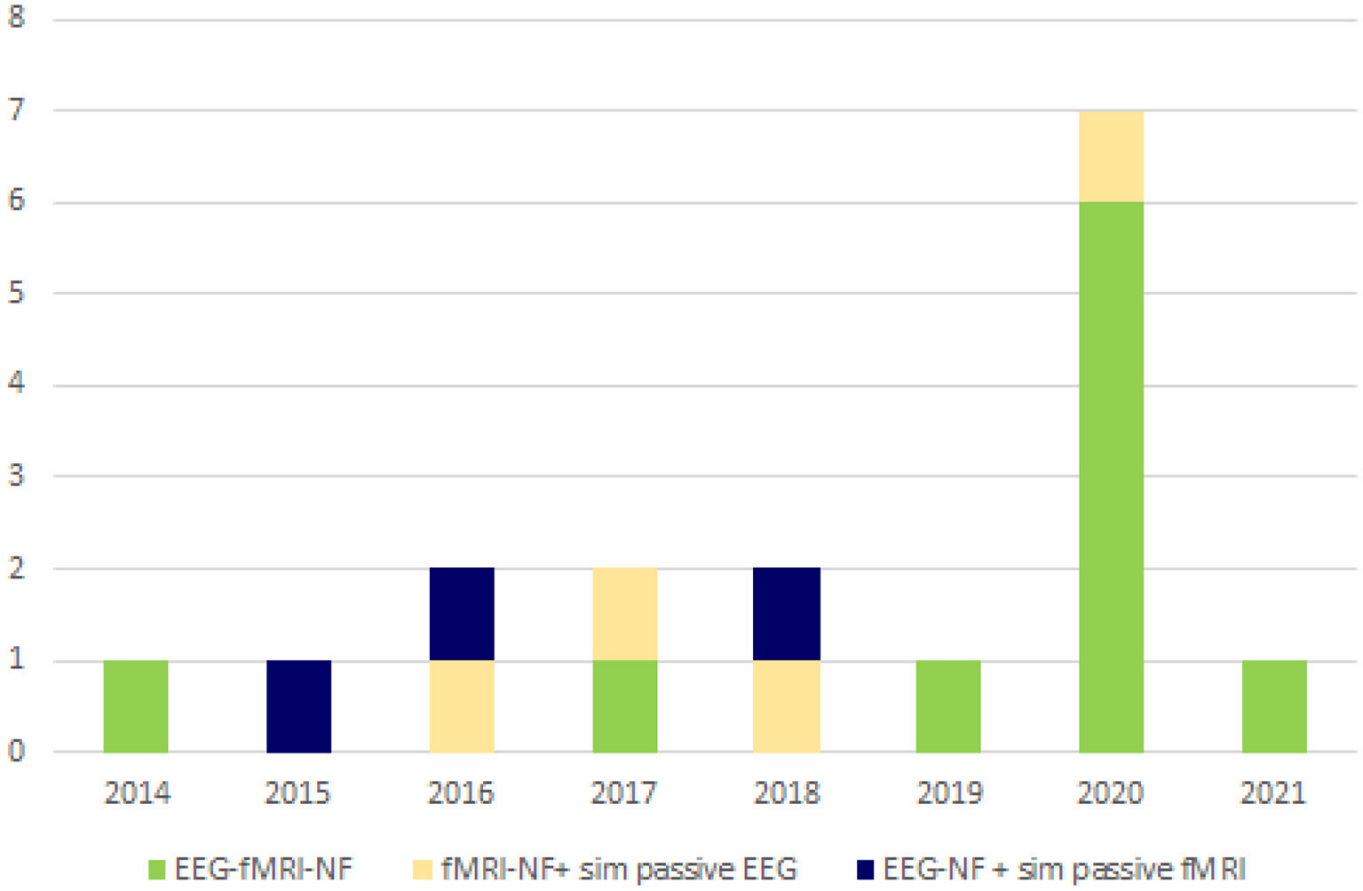
Distribution over the years of EEG-fMRI-NF studies. EEG-fMRI-NF research began surging in 2014; primary research continues to rise. This graph presents the composition of the publications found in our literature search. NF, neurofeedback; EEG, electroencephalograph; fMRI, functional magnetic resonance imaging.

**Figure 4:**
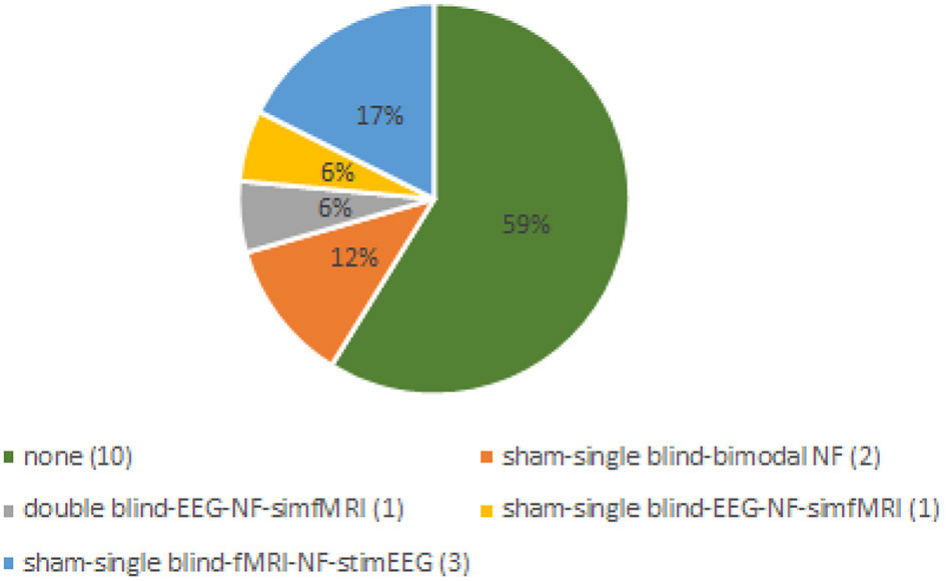
Distribution of controls employed in the review's studies. Sham neurofeedback is used to describe non-contingent feedback method. We consider controls absent if all participants of the studies received genuine feedback. With single blind we intend that participants of control groups were not aware of that. In the double-blind study both participants and experimenters were blind to NF group allocation. NF, neurofeedback; EEG, electroencephalograph; fMRI, functional magnetic resonance imaging.

Four of the bimodal EEG-fMRI-NF studies involved healthy participants without employing a control group (CG) or a control condition (Zotev et al., 2014; Perronnet et al., 2017; Cury et al., 2020; Lioi et al., 2020b) (Figures 3, 4). The first-ever approach of multimodal EEG-fMRI-NF was introduced by Zotev et al. The complex implementation of the bimodal real-time system included a General Electric Discovery MR750 whole-body 3 T MRI scanner and an MR-compatible EEG from Brain Products. Specifically, the system used a real-time fMRI feature reading software (AFNI) and a real-time BrainVision Recview EEG software. Real-time export of both signals was sent through a TCP/IP socket. Control and communication programs were scripted in Phyton and ran on a Linux computer. The fMRI BOLD was updated every TR from a selected ROI, whilst the EEG is updated faster from individual or group of channels (F3 and F4 electrodes in this paper), and real-time FFT spectrum analysis was computed for specific frequency bands. The multimodal NF graphical user interface (mGUI) integrated both EEG and fMRI signal, converting them into a graphical representation shown to the subject, which could be colored bars, text, or dynamic images. The aim of this study was to train the left amygdala [i.e., a sphere of 7 mm radius centered at (−21, −5, −16) in the Talairach space] and frontal EEG power asymmetries in the beta frequency band emotional through emotion self-regulation in healthy controls (HC) (Zotev et al., 2014). The results confirmed the feasibility of this multimodal NF technique which can then be implemented in therapeutic treatments for major depressive disorders. Perronnet et al. (2017) made the first comparison of unimodal fMRI-NF and EEG-NF during a motor imagery task versus bimodal EEG-fMRI-NF. The main aim was to determine the additional value of bimodal NF, hence the simultaneity of this hybrid system. The simultaneous system used MR-compatible EEG BrainVision and 3T MR. Feedback and visual instructions were transmitted using the Nordic Neurolab hardware and presented to the participant via an LCD screen and a rear-facing mirror fixed on the coil. This multimodal system is the same one used by Mano et al. (2017). Eighteen scalp channels on the primary motor area were used for EEG analysis, and the following Brodmann areas were used for fMRI: 44, 40, 47, 2 and 42. *Post-hoc* fMRI analysis showed that motor activations in the primary motor cortex (M1) were substantially greater during EEG-fMRI-NF than during EEG-NF. These areas are known to be involved in sensorimotor processes and visuospatial reasoning (e.g., Federico et al., 2022; Osiurak et al., 2022). They also found similar results of the EEG and fMRI of the emotional task study by Zotev et al. (2014). Perronnet et al. in this study also proposed a novel feedback metaphor for the bimodal NF that merges both EEG and fMRI signals into one single two-dimensional (2D) feedback. Another study focused on the possibility of computing and detecting fMRI-NF or EEG-fMRI-NF scores in motor imagery tasks, from EEG signals only (Cury et al., 2020). They compared different NF predictors, computed through a mathematical model, aiming to extract NF scores from the EEG-fMRI-NF data recorded from a pre-existing database previously published by Perronnet et al. (2017). The major goal of this study was to develop a system that can use only EEG to predict an NF score comparable to the NF score that might be obtained with a concurrent NF-EEG-fMRI session. The strategy was based on machine learning. To calculate and synchronize NF-EEG and NF-fMRI scores in real-time during a training phase, EEG and fMRI were both gathered at the same time. The resulting scores were then combined. Lioi et al. (2020b) validated a multimodal brain imaging data structure (BIDS) dataset of EEG-fMRI-NF during an imagery motor task to improve the learning of fMRI-informed EEG and the coupling mechanisms underlying both NF modalities. Such results emphasize how brain areas related to motor and visuospatial functions (e.g., Federico et al., 2021, 2022; Osiurak et al., 2022) may benefit significantly from using EEG-fMRI-NF. The proposed structure included in the format info relates to acquisition procedures, analyses, de-noising operators, and NF scores. Moreover, Mano et al. (2017) aimed to set up a hybrid EEG and fMRI platform for bimodal NF experiments, outlining all the steps required to build a multimodal NF system. It guides researchers in choosing software, hardware, experimental protocols, neurofeedback presentation and calculation.

### 3.3. Multimodal EEG-fMRI-NF on clinical population

Two bimodal EEG-fMRI-NF studies enrolled unmedicated patients affected by major depressive disorder (MDD) (Zotev et al., 2020; Zotev and Bodurka, 2020), both including a CG (Figures 3, 4). Zotev et al. (2020) evaluated EEG power asymmetries in frontal alpha and beta bands (FAA, FBA) during self-emotion regulation through autobiographical memories. Changes in EEG activities correlated with neural activations, and activations in prefrontal regions were associated with motivation and happiness (Davidson, 1995). Similarly, left amygdala BOLD activations were associated with FAA/FBA (Zotev et al., 2016). Therefore, simultaneous NF modulation of BOLD and FAA/FBA in MDD patients showed significant clinical improvements. The same research group (Zotev and Bodurka, 2020) investigated the effects of bimodal NF with EEG source activations through exact low-resolution brain electromagnetic tomography (eLORETA). The eLORETA values were computed using MNI coordinates of the EEG electrodes arranged according to the international 10–20 system. FFT power spectrum and consequent eLORETA were calculated for each given condition (happy memories, count and rest) in individual alpha frequency bands and for each run. Similar associations were found in EEG source density over prefrontal regions. MDD patients showed a large reduction of the upper alpha current source density in the left prefrontal region, associated with approach motivation. Concurrently, a significant reduction of high beta current density was observed in the right prefrontal region, indicating a reduction in avoidance-motivation related to anxiety (Zotev and Bodurka, 2020). Three studies enrolled stroke patients (Savelov et al., 2019; Lioi et al., 2020a; Bezmaternykh et al., 2021). Bezmaternykh et al. used a hybrid EEG-fMRI system and the OpenNFT suite for real-time estimation of the feedback. The ROIs chosen for fMRI were in the premotor and supplementary motor regions; EEG target was alpha and beta band in C3 and C4 electrodes (Bezmaternykh et al., 2021). Lioi et al. used the integrated EEG-fMRI-NF platform introduced by Mano et al. (2017), computing NF signal with Matlab. EEG 8–30 Hz frequency range was considered, and primary and supplementary motor areas were the fMRI ROIs (Lioi et al., 2020a). Savelov et al. referred to the hybrid platform (Mano et al., 2017), targeting BA 4 and certain EEG frequency bands which are not specified. In all cases, the clinical benefit derived from EEG-fMRI-NF training has been demonstrated, particularly regarding symptom improvement and gain of function. Simultaneous EEG-fMRI-NF training during motor imagery tasks of the affected limbs demonstrated clinical efficacy for motor improvement. In detail, NF treatment elicited activation in the ipsilesional supplementary motor area (SMA) and primary motor cortex (M1), according to the severity of the brain injury.

### 3.4. Unimodal fMRI-NF with passive simultaneous EEG

Four studies employed the fMRI-NF with simultaneous passive EEG recording, aiming to evaluate the electrophysiological activity deriving from the EEG signal that correlates with the hemodynamic active changes during a NF task. In two of these four studies, participants were HC (Zotev et al., 2018a; Simões et al., 2020), and the other two studies included groups of patients with MDD and post-traumatic stress disorder (PTSD) (Zotev et al., 2016, 2018b). Only three studies employed a control group (Zotev et al., 2016, 2018a,b), and apart from the PTSD paper, the other two studies show a statistical significance between EG and CG. Simões et al. (2020) investigated the possibility of reconstructing fMRI BOLD activity in the facial expressions processing network (FEPN) from simultaneous EEG recording in HC. The authors used the MR-compatible EEG NeuroScan SynAmps2 system, and the MaglinkTM software, and seven EEG source features were extracted on the electrodes in proximity to the FEPN in theta, low beta, high beta, gamma, and broadband). The extraction process of the EEG features was done through the independent component analysis, thus creating the FeatPoolICA-source model to predict the BOLD fMRI signal in FEPN. Studying the correlations between fMRI-NF BOLD activations and passive simultaneous EEG recording gave the possibility of shedding light on the neural networks underpinning cortical activations. Positive posterior alpha EEG power correlations with BOLD fluctuations was found in the mediodorsal (MD) and anterior (AN) thalamic nuclei, during a happy emotion induction task. These nuclei are involved in many functions, such as memory, emotion, motivation, learning, and decision-making (Zotev et al., 2018a). Zotev et al. (2016, 2018b) investigated correlations between FAA changes and the amygdala BOLD laterality in MDD patients upon emotion training (Zotev et al., 2016). FAA and alpha EEG coherence had a positive correlation with the left amygdala in PTSD patients (Zotev et al., 2018b), therefore showing significant reductions in the total mood disturbance. In all these three studies Zotev et al. used an EEG-fMRI system configuration where EEG was simultaneous but passive, i.e., no EEG information was used for real-time experiments. The target fMRI ROIs were defined as 14-mm diameter spheres in the Talairach space, and EEG electrodes used were F3, F4, F7, and F8. fMRI-NF was implemented using the custom real-time fMRI system utilizing the real-time functionality of AFNI as described previously (Zotev et al., 2011).

### 3.5. Unimodal EEG-NF with simultaneous passive fMRI

In three papers, fMRI was used as a passive simultaneous recording during EEG-NF training in HC. Two of them (Meir-Hasson et al., 2016; Keynan et al., 2019) applied algorithms to simultaneous EEG-fMRI-NF data to create an EEG prediction model called the electrical fingerprint (EFP), useful to probe the BOLD in the amygdala, thus relieving the need of a prior fMRI. They both enrolled HC with a CG, although Meir-Hasson et al. (2016) employed a relaxation task in HC, while Keynan et al. (2019) used an emotion-inducing (happiness) task in combat soldiers, which led to better stress coping. This framework showed that by using a single electrode in a single area, it was possible to create an EEG-EFP-NF model to predict BOLD activity. The effects of the feedback generated by this model were evaluated, and participants were able to modulate the activities resulting in a reduction of anxiety. The approach suggested in the paper by Meir-Hasson et al. used a regression model based on a representation of the EEG signal from a single channel in terms of time, frequency, and delay. The BOLD signal in the regions of interest was well estimated by the results, although the use of fMRI neurofeedback in this study simply supported the paradigm. The technique was intended to target the amygdala during NF-EEG sessions. Zich et al. (2015) instead applied EEG-NF with simultaneous passive fMRI during motor imagery tasks. The aim of the study was to investigate the concurrent BOLD correlating changes in the sensory-motor cortices upon EEG-NF training during a motor imagery task. Movement imagination results are like movement execution ones, and it showed that a complex relationship exists between sensorimotor cortex BOLD activations and scalp EEG signal, namely the SMR. These motor imagery-related findings support the usefulness of EEG-NF for motor recovery.

## 4. Discussion

The combination of new neuroimaging and electrophysiological techniques has encouraged an emerging field of research, namely the simultaneous application of EEG and fMRI in neurofeedback (EEG-fMRI-NF). This novel hybrid NF system takes advantage of the high spatial resolution of fMRI and the high temporal resolution of the EEG. Whilst unimodal NF is known to be a promising non-invasive therapeutic tool in neurological/psychiatric disorders, the multimodal EEG-fMRI-NF approach has been poorly explored so far. Therefore, there are still doubts about the clinical significance of these complementary measures on cognitive and behavioral performances. The present systematic review evaluated 17 EEG-fMRI NF studies in the literature according to their methodological characteristics as well as their clinical and neurophysiological outcomes. Such studies were heterogeneous in their design, methodology and application. Nine of these studies involved healthy participants (HC; Zotev et al., 2014, 2018a; Zich et al., 2015; Meir-Hasson et al., 2016; Perronnet et al., 2017; Keynan et al., 2019; Cury et al., 2020; Lioi et al., 2020b; Simões et al., 2020), while seven studies enrolled patients (Zotev et al., 2016, 2018b, 2020; Savelov et al., 2019; Lioi et al., 2020a; Zotev and Bodurka, 2020; Bezmaternykh et al., 2021).

Studies involving patients included small sample sizes and quite a compact clinical field of application, principally psychiatric. Therefore, such studies may appear not very well suited for detecting mild or moderate effects. Hence, future research should require larger samples to study NF effects in clinical populations. Eight papers explored the amygdala through emotional imagery (usually retrieving happy autobiographical memories), thus proving the potential use of EEG-fMRI-NF in MDD and PTSD (Zotev et al., 2014, 2016, 2018a,b, 2020; Keynan et al., 2019; Simões et al., 2020; Zotev and Bodurka, 2020). Interestingly, some studies in the past two decades used the SMR-NF protocol to control and reduce seizure threshold in epileptic patients, hence reducing sensorimotor system excitability (Bernstein et al., 2021; Loriette et al., 2021). Critically, SMR-NF protocol has poorly been used with the advanced procedure as simultaneous EEG-fMRI-NF, resulting in little research in areas such as epilepsy, thus suggesting a need for further studies of EEG-fMRI-NF in a wider clinical population type.

Six studies employed a motor imagery-motor task to explore the motor function, which has been linked to the activation of the primary motor cortex and supplementary motor area (Zich et al., 2015; Perronnet et al., 2017; Cury et al., 2020; Lioi et al., 2020a,b; Bezmaternykh et al., 2021; Federico et al., 2021, 2022; Osiurak et al., 2022). It should be noted that motor function was investigated in stroke patients only in three studies, with small sample sizes (Savelov et al., 2019; Lioi et al., 2020a; Bezmaternykh et al., 2021). Also, the clinical criterions used for these patients were unclear (e.g., Lioi et al., 2020a) as patients were presented with different clinical features, such as the severity of the stroke and its symptoms, and the level of damage to the cortico-spinal tract. In addition, it was not specified how long after the stroke onset the NF training was performed, and, finally, it has not been proven whether the benefits of NF training were long-lasting. On these bases, further studies should consider longer follow-up periods and document NF clinical effects.

According to our data, for the majority of studies, a key methodological concern is the number of NF sessions. It is common to implement a fixed number of sessions based on the effect sizes of similar protocols or studies. However, a training goal could also be established regarding a particular performance pattern, such as the reduction of certain symptoms as measured by the results of clinical questionnaires. In such cases, the number of sessions cannot be determined before the intervention, as it might be required to monitor subjects' learning curves to individually adapt the number of training sessions. Hence, practical factors and learner-specific traits should be considered while choosing the NF protocol. However, neuroplasticity may contribute to NF training outcomes. Congruently, there is a wide intra- and inter-variability among subjects depending on the type of disease and subjects' premorbid characteristics, such as cognitive reserve. With the type of disease, it might be important to consider whether there is structural and organic damage causing the impairment, such as strokes studies (Savelov et al., 2019; Lioi et al., 2020a), or if there is only a functional impairment with no structural changes, such as MDD studies (Zotev et al., 2016, 2020; Zotev and Bodurka, 2020). This strictly influences the target areas to choose and hence which neural connections to train during NF sessions. Hence, it becomes difficult to choose a fixed number of sessions. Up to now, little is known about neurofeedback sessions within a certain time interval being more helpful for learning to self-regulate brain activity, and less is known, even regarding the length of an effective gap between training sessions. Further studies should evaluate structural and functional changes after NF training, aiming to demonstrate the clinical efficacy and effects based on the number of sessions. Such future studies might repeat fMRI, and EEG following the training, and introduce a questionnaire to assess premorbid subjects' characteristics (e.g., cognitive reserve, which may moderate the relationship between brain pathology and clinical outcomes; Rosenich et al., 2020).

In our data, CG and/or a sham condition are only rarely represented (i.e., 41% of the studies do not employ a CG) (Zotev et al., 2014; Zich et al., 2015; Perronnet et al., 2017; Savelov et al., 2019; Lioi et al., 2020a,b; Bezmaternykh et al., 2021), without following consensus on the reporting and experimental design of clinical and cognitive-behavioral neurofeedback studies (CRED-NF checklist; Ros et al., 2020). In a few studies, single-blinded CG showed clinical and NF change improvements that were not statistically different from EG (Zotev et al., 2016, 2020). The reason why this happened could be associated with a placebo effect deriving from a beneficial psychological impact of the regular interaction with the clinical team during the study. However, it is to be noted that the CRED-NF checklist was just recently issued (2020). Hence, some authors were unable to use them as a reference for their investigations.

Recent unimodal fMRI-NF studies, which used the high spatial resolution of fMRI, found that learned control of localized BOLD activity in the amygdala corresponds with better emotion regulation in healthy individuals and may reduce the symptoms of stress and mood disorders (Keynan et al., 2019). However, the MRI approach's scalability (accessibility and cost-effectiveness), particularly in clinical applications, is significantly hampered by cost. A few intrinsic limitations make the MRI not always feasible, such as its costs, noises, contraindications, and patient compliance (i.e., claustrophobia, metal implant, etc.). The combination of EEG and fMRI enables regulating of discrete and defined brain areas, including the amygdala or thalamic nuclei. It also enables the analysis of associated EEG activity. The use of simultaneous EEG-fMRI recording in the context of real-time clinical neurofeedback is a recent application that was first introduced, and its feasibility was demonstrated by Zotev et al. (2014), Mano et al. (2017), and Perronnet et al. (2017). The creation of a new class of data known as EEG-fMRI-NF data, like the dataset provided by Perronnet et al. (2017), is made possible by recent technologies (Mano et al., 2017) that synchronize both EEG and fMRI signals for real-time neurofeedback. Perronnet's research also demonstrated that employing both neurofeedback modalities simultaneously in EEG-fMRI-NF sessions enhances the quality of the sessions. Hence, the opportunity to recreate an EEG-fMRI-NF session in real-time when using EEG only would reduce the use of fMRI in neurofeedback.

To get beyond MRI limitations, such as its costs and often reduced feasibility, mainly in clinical settings, some studies focused on the possibility of predicting and detecting fMRI-NF values through complex machine learning processes. Indeed, to export fMRI BOLD information, different methods have been exploited, which simulate and predict BOLD in a specific region of interest by learning from an EEG signal recorded simultaneously inside the fMRI scanner. Hence, specific EEG prediction models have been proposed in some studies, considering EEG recording linked to simultaneous fMRI changes (Meir-Hasson et al., 2016; Keynan et al., 2019; Simões et al., 2020). In addition, NF sessions might be used as learning sessions to improve the prediction of NF-fMRI scores. To tailor the model more effectively to the subject or patient, each new bi-modal neurofeedback session might be added to the subject-specific model. Future studies should investigate such an aspect. Another promising approach is called EEG finger-print (EFP), a time-frequency representation of the EEG that corresponds to the BOLD activity in a specific location. Additionally, finding correlations between frontal EEG frequencies asymmetries and amygdala BOLD activity, implies that EEG-NF designed to boost FAA might be complementary with fMRI-NF that is amygdala-based (Meir-Hasson et al., 2016; Keynan et al., 2019; Simões et al., 2020). The amygdala's role in associative learning, decision-making, memory, and attention is widely known. Thus, more brain disorders such as schizophrenia, frontotemporal dementia, and attention deficit hyperactive disorder might be further investigated with the multimodal approach EEG-fMRI-NF, as done with unimodal one. Similar functions are solved also by other subcortical brain structures, such as the thalamus nuclei, which are involved in memory and cognitive roles. For instance, Bernstein et al. (2021) demonstrated the impairment of thalamus nuclei in the early stages of MCI and Alzheimer's disease, as recently shown in the clinical neurological literature (Ilardi et al., 2022). Zotev et al. (2018a) employed fMRI-NF with simultaneous passive EEG to modulate EEG and BOLD activity in these brain areas, resulting in a promising use of NF training in prodromal stages of neurodegenerative disease aiming to improve neuroplasticity.

Multimodal EEG-fMRI-NF has limitations in setting, as providing feedback with both modalities requires complex hardware and software installation and longer time preparation for the patient. Both raw data from EEG and fMRI need to go through some pre-processing steps before being shown to participants, to reduce the signal-to-noise ratio and guarantee accurate and precise feedback (e.g., remotion of gradient artifact, ballistocardiogram artifact, motion correction, features extraction) (Loriette et al., 2021). The simultaneity of the two modalities increases the cognitive load of the subjects when aiming to modulate both measures, also according to the feedback metaphor's choice (1D or 2D). Only Perronnet et al. (2017) evaluated different feedback metaphor interfaces. 1D merges EEG and fMRI information in one gauge as a single regulation task, like the thermometer, whilst the 2D feedback is a plot that sees the two modalities in two different dimensions. This approach shows a successful regulation of EEG and fMRI simultaneously with both strategies. The difference stands in the complexity of the neural networks recruited with the 1D or the 2D feedback. 1D results are easier to control hence it relieves the cognitive load of the subject, 2D instead has a complex representation, and it invites participants to seek out and investigate more specialized brain patterns to regulate both measurements increasing the cognitive load (Gaume et al., 2016). Certain studies revealed that NF efficacy on the learning curve depends more on EEG than fMRI modulation. This may be because EEG measure is more challenging to control and modulate, necessitating greater effort at fMRI expenses. Given the difference in modulating the two measures, the choice of the NF metaphor's interface depends on the clinical population included in the studies. However, it is only partially understood which substantial theoretical aspects may underpin the EEG-fMRI-NF learning processes. Therefore, a larger investigation and use of these technologies is required to improve the clinical use of such novel hybrid systems.

## 5. Conclusion

This review presents a compelling argument for using simultaneous EEG-fMRI-NF protocols. By combining EEG and fMRI, EEG-fMRI-NF may enhance brain rehabilitation techniques, enabling the volitional regulation of two complementary bio-signals, namely, the electric brain activity (EEG) and BOLD (fMRI). This systematic review shows potential therapeutic effects of EEG-fMRI coupling in NF training on certain brain regions, despite methodological issues, small sample sizes, and intrinsic heterogeneity limiting the generalizability of findings. However, given most studies use a single session of NF with either EEG, fMRI, or their combination, it is difficult to draw conclusions about the clinical efficacy of NF. Therefore, future studies are needed to fully understand both clinical and non-clinical potentialities of EEG-fMRI-NF.

## Data availability statement

The original contributions presented in the study are included in the article/supplementary material, further inquiries can be directed to the corresponding author.

## Author contributions

GF, CC, and GC contributed to conception and design of the work. GC performed the literature research and draft the manuscript. GF and GM reviewed and selected the articles to include in the systematic review. CRI, MM, ADC, and VA revised the drafts. MS performed manuscript supervision and project administration. All authors have read and agreed to the published version of the manuscript.
